# Maternal PlGF and sFlt-1 are Associated with Low Birth Weight and/or Small-for-Gestational Age Neonates in Pregnancy with or without Preeclampsia

**DOI:** 10.1055/a-2555-1742

**Published:** 2025-03-31

**Authors:** Wenbo Luo, Pingping Tang, Yan Lv, Yijun Song, Huiying Hu, Jinsong Gao, Juntao Liu

**Affiliations:** 1 Department of Obstetrics and Gynecology, Peking Union Medical College Hospital, Chinese Academy of Medical Sciences, Peking Union Medical College, National Clinical Research Center for Obstetric and Gynecologic Diseases, Beijing, China

**Keywords:** placental growth factor, soluble fms-like tyrosine kinase-1, low birth weight, small-for-gestational age

## Abstract

**Objective:**

To evaluate the association of PIGF and sFlt-1 with low birth weight and/or small-for-gestational age neonates in pregnancy with or without preeclampsia.

**Methods:**

Singleton pregnancies with sFlt-1/PlGF tested were included and set into four groups for case–control analysis. Distribution of sFlt-1/PlGF, sFlt-1, PlGF, and PGF% were evaluated, Kruskal–Wallis test and Mann–Whitney U test were adopted for significance analysis.

**Results:**

Maternal sFlt-1/PlGF, PlGF, PlGF%, and sFlt-1 were statistically associated with low birth weight and/or small-for-gestational age in pregnancy complicated or uncomplicated with preeclampsia. A significant difference was shown on sFlt-1/PIGF ( *p* = 0.0082), PIGF% ( *p* = 0.0326), PIGF ( *p* = 0.0128), and sFlt-1 ( *p* = 0.0469) in pregnancy with small-for-gestational age and/or low birth weight neonates. A significantly higher median of sFlt-1/PlGF (448 vs. 61.6, *p* < 0.0001) and sFlt-1 (15499 vs. 3226, *p* < 0.0001), a significantly lower median of PlGF (33.92 vs. 115.2, *p* < 0.0001) and PlGF% (−76.63 vs. −20.31, *p* < 0.0001) were demonstrated, respectively, when preeclampsia with small-for-gestational age and/or low birth weight neonates was compared with preeclampsia with normal birth weight neonates. No significant difference was demonstrated between low birth weight and small-for-gestational age on sFlt-1/PlGF, PlGF, PlGF%, and sFlt-1.

**Conclusion:**

sFlt-1/PlGF seems to be a promising biomarker in predicting low birth weight and/or small-for-gestational age neonates in pregnancy with or without preeclampsia.

Maternal and fetal health are of worldwide concern, and great efforts have been paid to reduce pregnancy-associated adverse outcomes with aim to safeguarding perinatal health. Preeclampsia is a common pregnancy-specific complication characterized by compromised placental function with a prevalence at 3 to 5%, leading to maternal mortality and preterm delivery, and the latter consequently contributes to low birth weight or small-for-gestational age neonates, which are pivotal contributors to neonatal deaths. 1 2 It is estimated that approximately 26.2% of neonates born worldwide are either preterm or small-for-gestational age or low birth weight, accounting for about 55.4% of global neonatal mortality. 2 3 Therefore, early evaluation of preeclampsia and the possibility of delivering small-for-gestational age or low birth weight neonates are of crucial importance to the improvement for perinatal health.

Maternal serum angiogenic markers like placental growth factor (PlGF) and soluble fms-like tyrosine kinase-1 to PlGF ratio (sFlt-1/PlGF) have been widely researched in its association with perinatal adverse outcomes, like preeclampsia, preterm birth, and fetal growth restriction. 4 5 6 7 8 9 Unlike low birth weight and small-for-gestational age clearly defined by neonate's birth weight, fetal growth restriction implies a pathological restriction of the genetic growth potential and may manifest evidence of fetal compromise, which is inherently unknown prenatally and hard to be clinically evaluated, making its clinical definition and diagnosis quite challenging and disputable. 10 11 A caveat existed that all fetal growth restrictions will be small-for-gestational age, but not all small-for-gestational age have been diagnosed as fetal growth restrictions; furthermore, not all small-for-gestational age are low birth weight, and not all low birth weight are small-for-gestational age. Regardless of the confusion, low birth weight and small-for-gestational age together covered all low weight-related problems of neonates and these two indicators are much easier and sharply to be clinically evaluated and directly correlate with neonatal medical care and mortality. Therefore, we set out to evaluate low birth weight and small-for-gestational age here as neonatal abnormal birth weight indicators.

A prior study revealed that PlGF < 100 ng/L or raised sFlt-1/PlGF ratio was strongly associated with increased risk for medically indicated preterm birth in both growth-restricted and appropriate-for-gestational age infants. 7 Though preterm birth is a main contributor to low birth weight and small-for-gestational age, however, the association of delivering low birth weight and/or small-for-gestational age neonates with maternal PlGF and sFlt-1/PlGF so far still remains elusive. In addition, Preeclampsia is commonly associated with delivering low birth weight and/or small-for-gestational age neonates, whether the expression of PlGF and sFlt-1 in preeclampsia are statistically associated with low birth weight or small-for-gestational age remains undiscussed as well.

Therefore, in this study we aimed to evaluate the relation of maternal PlGF and sFlt-1 with low birth weight and/or small-for-gestational age neonates in pregnancy with or without preeclampsia, and whether the correlation is significantly capable of supporting its possible use in predicting low birth weight and/or small-for-gestational age neonates.

## Materials and Methods

### Population in the Study

This retrospective study only included singleton Chinese pregnant women who had undergone sFlt-1/PlGF tests for at least once from 20 + 0 weeks of gestation to the delivery. All participants followed regular prenatal medical care and delivered their babies at our medical center from January 2022 to August 2023. Basic medical conditions during pregnancy, pregnancy outcomes, maternal age at delivery, pregnancy length, neonatal sex and neonatal birth weight were accurately derived from medical history.

### Criteria for Subgroups

According to pregnancy outcomes, four subgroups were set as follows: healthy pregnancy (Group A), pregnancy complicated with preeclampsia (Group B), other maternal diseases during pregnancy (Group C), pregnancy with small-for-gestational age or low birth weight neonates (Group D). Healthy pregnancy was defined as pregnancy without any known maternal and fetal complications and the birth weight of neonate within a normal range. The diagnostic criteria for preeclampsia were based on American College of Obstetrics and Genecology practice bulletin for gestational hypertension and preeclampsia, which defines preeclampsia as a new-onset hypertension with systolic blood pressure ≥140 mmHg and/or diastolic blood pressure ≥90 mmHg after 20 weeks of gestation, accompanied by at least one of new-onset conditions like proteinuria, impaired liver function, severe persistent right upper quadrant or epigastric pain and not accounted for by alternative diagnoses, renal insufficiency, pulmonary edema or new-onset headache unresponsive to acetaminophen and not accounted for by alternative diagnoses or visual disturbances. 12 13 Pregnancy delivering neonate with normal weight but complicated with chronic hypertension, gestational hypertension, diabetes, gestational diabetes, autoimmune disease like antiphospholipid syndrome and systemic lupus erythematosus, and chronic kidney diseases were included in Group C. 12 In group D, small-for-gestational age was defined as birthweight below the 10 ^th^ percentile for gestational age adjusted by sex and low birth weight referred to neonatal weight < 2500g. 2 14

### sFlt-1/PlGF Testing

For those who had undergone multiple sFlt-1/PlGF tests, only the latest sFlt-1/PlGF results before delivery were included in this study for analysis. sFlt-1/PlGF were measured by SuperFlex (PerkinElmer Inc) which is an automated point-of-care chemiluminescence analyzer with a linear range of 2 to 2706 pg/mL, commercial quality control samples in regents were used to assure its measurement performance. Besides sFlt-1/PlGF, sFlt-1 and PlGF, PIGF% was evaluated as the fourth metrics and PlGF% was calculated as the following: the measured concentration minus 5 ^th^ percentile concentration of the average pregnancy at the same gestational age, then divided by 5 ^th^ percentile concentration.

### Data Analysis and Statistics

First, descriptive statistics were employed to illustrate characteristics of different groups. Second, Kruskal–Wallis test was conducted to evaluate the significance of expression difference of sFlt-1/PlGF, PlGF, PlGF% and sFlt-1 among four groups. Third, Mann–Whitney U test was performed for significance evaluation within groups. Statistical significance level was set at *p* < 0.05. Graphpad Prism 8.0.2 and Excel 2016 were used for graph-making, tables and statistical analysis.

## Results

Two hundred fifty-five Chinese women with sFlt-1/PlGF measured at 20 + 0 to 40 + 0 weeks were analyzed in this study, of which fifty-nine were classified into healthy pregnancy (Group A), forty-five into pregnancy complicated with preeclampsia (Group B), one hundred into other diseases during pregnancy (Group C) and fifty-one into pregnancy with small-for-gestational age and/or low birth weight neonates (Group D). Table 1 shows the characteristics of participants in four groups. Pregnancy with preeclampsia is commonly accompanied by preterm birth, thirty out of forty-five (30/45, 66.67%) in Group B delivered either small-for-gestational age or low birth weight neonates, of which twenty-two (22/22, 100%) were in early-onset preeclampsia (preeclampsia diagnosed before 34 gestational weeks) and eight (8/23, 32.78%) were in late-onset preeclampsia. ( Table 2 )

**Table 1:** The demographic characteristics of participants included in four groups

Group	*N*	Expected delivery age	Gestational age at sampling	Gestational age at delivery	Preeclampsia diagnosed day
Group A	59	34.28 ± 4.47	205.4 ± 31.33	273.6 ± 8.38	
Group B	45	33.44 ± 3.84	220.2 ± 27.84	240.7 ± 25.30	236.1 ± 25.94
Early-onset preeclampsia	22	33.23 ± 4.67	210.1 ± 20.19	219.2 ± 18.63	210.8 ± 19.69
Late-onset preeclampsia	23	33.64 ± 2.93	229.9 ± 31.01	261.3 ± 6.64	256.4 ± 8.73
Group C	100	35.03 ± 4.00	210.9 ± 33.95	269.7 ± 8.90	
Diabetes	37	35.81 ± 4.25	204.4 ± 32.27	271.1 ± 7.90	
Hypertension	13	36.43 ± 4.85	216.7 ± 39.12	273.7 ± 5.51	
Autoimmune disease	18	34.01 ± 3.36	210.6 ± 27.15	266.1 ± 10.87	
At least two of the above	32	34.14 ± 3.45	216.3 ± 37.17	268.6 ± 9.28	
Group D	51	34.15 ± 4.33	208.3 ± 27.64	258.6 ± 20.97	
Low birth weight	12	35.46 ± 3.93	193.8 ± 29.72	238.6 ± 15.47	
Small-for-gestational age	22	33.44 ± 4.23	207.0 ± 28.81	274.2 ± 4.30	
Both of the above	17	34.16 ± 4.82	219.4 ± 21.48	252.3 ± 21.84	

**Table 2:** Distribution characteristics and significance by Mann–Whitney U test of sFlt-1/PlGF, PlGF, PlGF%, and sFlt-1

Groups	Subgroups	*N*	sFlt-1/PlGF		PlGF		PlGF%		sFlt-1	
			Median (5th–95th)	*p*	Median (5th–95th)	*p*	Median (5th–95th)	*p*	Median (5th–95th)	*p*
Preeclampsia	Early onset	22	546.4 (15–6,424)	<0.0001	32 (3.64–203.2)	<0.0001	−78.4 (−97.1 to 50.24)	<0.0001	19159 (1,227–66954)	<0.0001
	Late onset	23	89.99 (0.76–846.5)		83.79 (23.89–931.9)		−29 (−80.8 to 845.8)		4,227 (637.4–30,358)	
Preeclampsia	Neonate with abnormal weight	30	448 (9.56–5533)	<0.0001	33.92 (4.79–146)	<0.0001	−76.63 (−95.9 to 12.62)	<0.0001	15,499 (1,382–58,071)	0.0001
	Neonate with normal weight	15	61.6 (0.7–179.5)		115.2 (44.5–1022)		−20.31 (−75.8 to 769.9)		3,226 (452.7–14,952)	
Neonate with abnormal birth weight	Low birth weight	12	4.905 (1.49–127.3)	0.511	169.4 (30.8–536.9)	0.261	62.14 (−79.24 to 569)	0.4026	2,471 (570.7–6,321)	0.3092
	Small-for-gestational age	22	5.505 (0.58–107.2)		250.2 (38.45–1126)		117.6 (−73.4 to 665.8)		1,810 (336.6–8,773)	

**Fig. 1:**
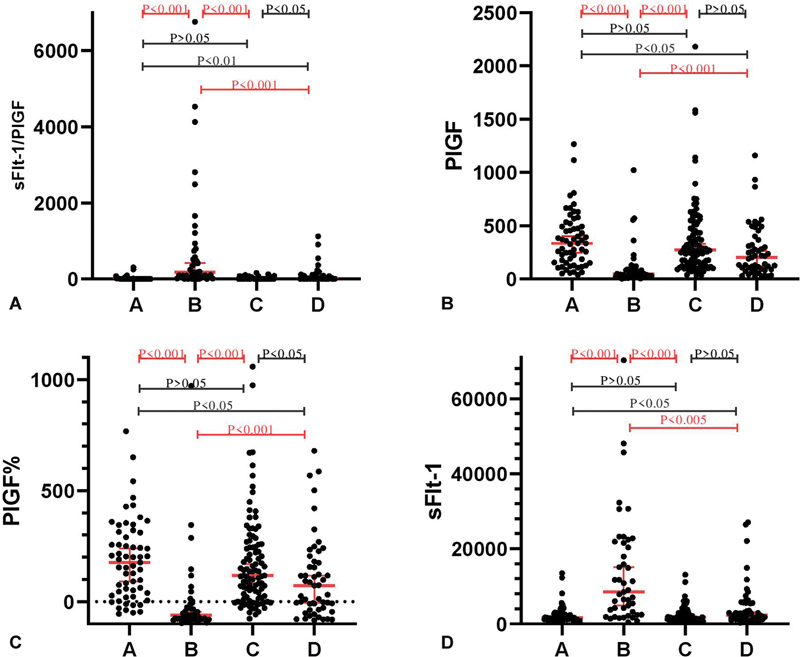
Distribution of sFlt-1/PlGF, PlGF, PlGF%, and sFlt-1 in four groups. Group A, B, C, and D represented healthy pregnancy, pregnancy complicated with preeclampsia, pregnancy coexisting with other maternal diseases like chronic hypertension, diabetes, gestational diabetes, and autoimmune disease, and pregnancy with small-for-gestational age or low birth weight neonates, respectively.

Distribution characteristics of sFlt-1/PlGF, PlGF, PlGF% and sFlt-1 in four groups ( Table 3 , Fig. 1 ) demonstrated overlapping in concentration expression of sFlt-1/PlGF, PlGF, PlGF% and sFlt-1 on all four groups even though a significant difference existed between the medians. For sFlt-1/PlGF, Group B presented the highest median (179.5) and was seconded by Group D (7.96), Group C (4.65) and Group A (3.64). For PlGF, Group B presented the lowest median (48.28) and was seconded by Group D (204), Group C (271.9) and Group A (335.4). PIGF% presented the same changing trend with PIGF and sFlt-1 followed that of sFlt-1/PIGF.

**Table 3:** Distribution characteristics of sFlt-1/PlGF, PlGF, PlGF%, and sFlt-1 in case–control groups

		Healthy pregnancy ( *N* = 59)	Preeclampsia ( *N* = 45)	Pregnancy with complications ( *N* = 100)	Fetus diagnosed with low birth weight or small-for-gestational age or both ( *N* = 51)
sFlt-1/PlGF	Median	3.64	179.5	4.65	7.96
	5th percentage	1.09	1.348	1.31	0.98
	95th percentage	97.57	4410	85.1	688.8
	Mean	18.51	739.7	17.08	90.83
PlGF (pg/mL)	Median	335.4	48.28	271.9	204
	5th percentage	56.9	6.9	72.62	28.66
	95th percentage	806.4	565.9	1099	892.7
	Mean	364	109	364.3	261.3
PlGF%	Median	176.5	−61	117.8	72.69
	5th percentage	−45.19	−94.7	−30.87	−79.67
	95th percentage	542.3	327.6	667.8	576
	Mean	187.5	−7.711	184.5	105.3
sFlt-1 (pg/mL)	Median	1,627	8,516	1,730	2,393
	5th percentage	711.5	957	702.5	624.2
	95th percentage	8,112	47365	6180	23,827
	Mean	2,385	13,744	2,428	4,681

According to Kruskal–Wallis test, Group B showed a significant difference on sFlt-1/PlGF, PlGF, PlGF% and sFlt-1 with Group A, Group C and Group D with all *p* < 0.0001, and no statistical significant difference was demonstrated between Group A and Group C. (shown in Table 4 ) These strengthened the evidence that diseases like diabetes, hypertension and/or autoimmune diseases performed no impact on sFlt-1/PlGF, PlGF, PlGF% and sFlt-1, and sFlt-1/PlGF, PlGF, PlGF% and sFlt-1 are very helpful biomarkers in differentiating preeclampsia from healthy pregnancy and pregnancy complicated with diabetes, hypertension and/or autoimmune diseases. Group A showed a significant difference with Group D on sFlt-1/PIGF ( *p* < 0.01), PIGF% ( *p* < 0.05), PIGF ( *p* < 0.05) and sFlt-1 ( *p* < 0.05), indicating that sFlt-1/PlGF, PlGF, PlGF% and sFlt-1 may be capable as biomarkers, especially sFlt-1/PlGF ( *p* < 0.01), in distinguishing pregnancy ending up with small-for-gestational age and/or low birth weight neonates from healthy pregnancy.

**Table 4:** Significance evaluation among groups by Kruskal–Wallis test

	*p* -Value by Kruskal–Wallis			
Comparison in pairs	sFlt-1/PlGF	PlGF	PlGF%	sFlt-1
Group A vs. Group B	<0.0001	<0.0001	<0.0001	<0.0001
Group A vs. Group C	>0.05	>0.05	>0.05	>0.05
Group A vs. Group D	0.0082	0.0326	0.0128	0.0469
Group B vs. Group C	<0.0001	<0.0001	<0.0001	<0.0001
Group B vs. Group D	<0.0001	<0.0001	<0.0001	0.0004
Group C vs. Group D	0.0267	0.0682	0.0399	0.0583

For preeclampsia is commonly accompanied by preterm birth which further results in low birth weight or small-for-gestational age neonates, so to figure out the impact of neonatal birth weight on maternal PIGF and sFlt-1 in preeclampsia, Group B was further divided into two subgroups according to whether or not having a small-for-gestational age and/or low birth weight neonate. It turned out that small-for-gestational age and/or low birth weight had a significant impact on sFlt-1/PlGF, PlGF, PlGF% and sFlt-1 with all *p* < 0.0001 in pregnancy complicated with preeclampsia. (shown in Table 2 ) When early-onset preeclampsia was compared to late-onset preeclampsia and preeclampsia with small-for-gestational age and/or low birth weight neonates was compared to preeclampsia with normal birth weight neonates, a significantly higher median of sFlt-1/PlGF (546.4 vs. 89.9, 448 vs. 61.6, *p* < 0.0001) and sFlt-1 (19159 vs. 4227, 15499 vs. 3226, *p* < 0.0001), a significantly lower median of PlGF (32 vs. 83.79, 33.92 vs. 115.2, *p* < 0.0001) and PlGF% (−78.4 vs. −29, −76.63 vs. −20.31, *p* < 0.0001) were demonstrated, respectively. ( Table 2 ). These implied a higher sFlt-1/PlGF or sFlt-1, or a lower PlGF or PlGF% can as well refer to a higher possibility in having a low birth weight and/or small-for-gestational age neonate in diagnosed preeclampsia pregnancy. In other words, sFlt-1/PlGF, PlGF, PlGF% and sFlt-1 can not only be biomarkers of preeclampsia subtyping but also biomarkers of low birth weight and/or small-for-gestational age neonates in preeclampsia pregnancy.

In Group D, sFlt-1/PlGF, PlGF, PlGF% and sFlt-1 of low birth weight neonates were further compared with these of small-for-gestational age neonates by Mann–Whitney U test, no significant difference was demonstrated with all P > 0.05 ( Table 2 ), suggesting low birth weight and small-for-gestational age had an equal impact on sFlt-1/PlGF, PlGF, PlGF% and sFlt-1 and thus can be evaluated together. Similarly, in Group C, no statistical difference was shown among pregnancy only complicated with diabetes, or with hypertension or with autoimmune diseases on the expression of sFlt-1/PlGF, PlGF, PlGF% and sFlt-1 through Kruskal–Wallis test. Group C and Group D presented a significant difference only on sFlt-1/PlGF and PlGF% ( *p* < 0.05) but not on PlGF and sFlt-1 ( Table 4 ). All of the above implies if pregnancy is complicated with diabetes, hypertension and/or autoimmune diseases, sFlt-1/PlGF and PlGF% rather than PlGF and sFlt-1 alone should be preferred as biomarkers in helping screening out pregnancy that will deliver small-for-gestational age and/or low birth weight neonates.

## Discussion

In this retrospective case–control analysis, we found out that sFlt-1/PlGF, PlGF, PlGF% and sFlt-1 are statistically associated with low birth weight and/or small-for-gestational age with *p* < 0.05 in pregnancy complicated or uncomplicated with preeclampsia, and sFlt-1/PlGF showed a more significant difference with *p* < 0.01, meaning sFlt-1/PlGF is a better indicator in distinguishing pregnancy that will deliver low birth weight and/or small-for-gestational age neonates. These are consistent with Rahman's prospective cohort study 15 which claimed PlGF and sFlt-1/PlGF may be useful second-trimester biomarkers for small-for-gestational age. While taking into account that the difference of sFlt-1/PlGF, PlGF, PlGF% and sFlt-1 are much stronger between Group A,C,D and Group B ( *p* < 0.0001) than between Group A and Group D ( *p* < 0.05), the clinical utility of using sFlt-1/PlGF, PlGF, PlGF% and sFlt-1 to predict pregnancy that will deliver low birth weight and/or small-for-gestational age neonates out of healthy pregnancy will be worse than predicting preeclampsia out of healthy pregnancy or pregnancy that will deliver low birth weight/ small-for-gestational age neonates. Further prospective studies are needed to evaluate and compare its clinical efficiency.

A significant difference was demonstrated on sFlt-1/PlGF (448 vs. 61.6, *p* < 0.0001), PlGF (33.9 vs. 115.2, *p* < 0.0001), PlGF% (−76.6 vs. −20.3, *p* < 0.0001), and sFlt-1 (15499 vs. 3226, *p* < 0.0001) in preeclampsia with low birth weight and/or small-for-gestational age neonates or with normal birth weight and/or appropriate-for-gestational age, demonstrating sFlt-1/PlGF, PlGF, PlGF%, and sFlt-1 can also be biomarkers of low birth weight and/or small-for-gestational age neonates in diagnosed preeclampsia pregnancy. To the knowledge, the only cure of preeclampsia to safeguard maternal safety is delivery which is at the cost of having an immature neonate, predicting low birth weight and small-for-gestational age neonates can be helpful for neonatal care after delivery. However, cutoffs of sFlt-1/PlGF, PlGF, PlGF%, and sFlt-1 are not studied in this analysis and further efforts are needed.

Pregnancy with low birth weight neonates and pregnancy with small-for-gestational age neonates were compared in this study and showed no significant difference on sFlt-1/PlGF (4.9 vs. 5.5, *p* = 0.51), PlGF (169.4 vs. 250.2, *p* = 0.26), PlGF% (62.1 vs. 117.6, *p* = 0.40), and sFlt-1 (2471 vs. 1810, *p* = 0.31). This finding which has not been revealed before paves the evidence that low birth weight and small-for-gestational age had the same impact on sFlt-1/PlGF, PlGF, PlGF%, and sFlt-1 and can be evaluated together as birth weight abnormality markers.

The significant difference between Group B and Group C, Group B and Group D strengthened the basis for clinical practice that sFlt-1/PlGF, PlGF, PlGF%, and sFlt-1 can effectively distinguish preeclampsia from pregnancy complicated with hypertension, diabetes, immune diseases, chronic kidney diseases, and those will deliver low birth weight/ small-for-gestational age neonates. These to some degrees echoed with Nir Melamed's finding 16 that sFlt-1 and PlGF can effectively predict preterm preeclampsia among patients with chronic kidney disease, and Provendier's research 17 that sFlt-1/PlGF presented an area under the curve of 0.90 in prediction preeclampsia in women with preexisting diabetes during the third trimester.

Apart from the above findings, in this study, we evaluated not only sFlt-1/PlGF, PlGF, and sFlt-1 but also PlGF% as biomarkers associated with low birth weight and/or small-for-gestational age. Unlike PlGF alone with its cut-off usually set at 100pg/mL, 5 ^th^ PlGF was a metric less being researched which may attribute to a lack of self-established PlGF reference range adjusted by gestational age. 18 However, compared with PlGF, 5 ^th^ PlGF functions as a better indicator than PlGF with a certain cutoff alone under some circumstances. Meanwhile, distribution of sFlt-1/PlGF, PlGF, PlGF%, and sFlt-1 in groups were demonstrated and compared for clinical and laboratory specialists to get an overall glimpse of the changing trends of maternal angiogenic biomarkers in different maternal and neonatal circumstances. There are shortcomings to be improved as well. First, maternal plasma sampling time was not accurately standardized but limited in a time window ranging from 20 + 0 gestational weeks to delivery. Second, this was a retrospective analysis based on real-world laboratory data with a limited size in single medical center. Third, clinical utility of sFlt-1/PlGF, PlGF, PlGF%, and sFlt-1 was not evaluated and compared. Regardless of these shortcomings, this retrospective analysis still revealed that sFlt-1/PlGF, PlGF, PlGF%, and sFlt-1, especially sFlt-1/PlGF and PlGF%, have the potential in predicting low birth weight and small-for-gestational age neonates in pregnancy with or without preeclampsia.
